# Monocomponent chemoembolization in oral and oropharyngeal cancer using an aqueous crystal suspension of cisplatin

**DOI:** 10.1038/sj.bjc.6600042

**Published:** 2002-01-21

**Authors:** A F Kovács, P Obitz, M Wagner

**Affiliations:** Clinic for Maxillofacial Plastic Surgery, Johann Wolfgang Goethe-University Medical School, Theodor-Stern-Kai 7, 60590 Frankfurt am Main, Germany; Department of Pharmacy, Johann Wolfgang Goethe-University Medical School, Theodor-Stern-Kai 7, 60590 Frankfurt am Main, Germany; Institute for Pathology, University of Essen Medical School, Hufelandstraße 55, 45122 Essen, Germany

**Keywords:** head and neck neoplasms, chemoembolization, cisplatin, crystallization, suspensions

## Abstract

Intensification of intra-arterial chemotherapy with high-dose cisplatin and concomitant reduction of toxicity under the conditions of the head and neck was aimed at by combination of antineoplastic activity and embolizing effect in the same pharmacon. A cisplatin suspension in normal saline (5 mg in 1 ml) with precipitation of microembolizing cisplatin crystals was prepared. No additional pharmacons. Cisplatin dosage was 150 mg m^−2^, maximum absolute dose 300 mg, maximum amount of fluid 60 ml. Thirty patients (UICC-Classification of tumours: I/2 patients, II/6, III/2; IV/20) were treated in a neoadjuvant setting with superselective chemoembolization using the cisplatin suspension. A control group (*n*=30) with the same tumour and nodal staging was treated with a usual cisplatin solution (150 mg m^−2^ dissolved in 500 ml saline). In both groups, parallel intravenous infusion of sodium thiosulphate (9 g m^−2^). Endpoints were toxicity and response. Continuation of treatment by surgery or radiation. Overall remission was 70% in the study group and 46.7% in the control group after one cycle respectively. Systemic side-effects were very low (grade I WHO) in both groups. Side-effects were found to be similar to post-embolization syndrome (swelling, mild to moderate pain, leucocytosis without fever) in the study group. Chemoembolization in the head and neck area can be carried out routinely using this method.

*British Journal of Cancer* (2002) **86**, 196–202. DOI: 10.1038/sj/bjc/6600042 www.bjcancer.com

© 2002 The Cancer Research Campaign

## 

Regional chemotherapy via a tumour-feeding artery achieves higher drug exposure at the target site than with corresponding systemic exposure and, therefore, has the possibility of generating higher therapeutic response without jeopardizing tolerance. In head and neck squamous cell carcinoma (SCC) patients, Robbins *et al* (1994) found a maximum tolerated dose of intra-arterially delivered cisplatin of 150 mg m^−2^. Cisplatin was chosen because sodium thiosulphate can be used as systemic antagonist. If thiosulphate circulates intravenously, cisplatin becomes chelated and inactivated (Pfeifle *et al*, 1985). By this method, plasma clearance is increased and drug targeting becomes still higher. Sensitive compartments, such as the bone marrow, gut, and kidneys are protected from the toxic effect of the drug. Using modern small catheter systems via the femoral artery, it was possible to infuse the antineoplastic drug superselectively (mainly into the lingual or facial artery). These small vessels have a low flow (about 120 ml min^−1^) which additionally raises drug targeting. This therapeutic approach in head and neck SCC showed good results with respect to response and toxicity (*n*=>300 patients (Kovács *et al*, 1999; Robbins *et al*, 2000). Another method of increasing the regional advantage is the reduction or stop of blood flow using microcapsules (Kato *et al*, 1996) or embolizing agents (Araki *et al*, 1989) resulting in longer tumour residence time, enhancement of first-pass extraction of the drug, and also hypoxic necrosis which is intended. Embolization with encoated drug microcapsules or mixtures of drugs and embolizing agents were mainly used in liver tumours because the liver is capable of compensating local necrosis due to the fact that most of the metastases gain their blood supply via the hepatic artery (Breedis and Young, 1954) while the rest of tissue is nourished by the portal vein. Besides the drawback of a complicated technical production of embolizing agents, there are extreme obstacles concerning this method of a complete temporary halt of blood flow in the head and neck area: intolerable local necrosis and danger to eyes and nerve ganglions via anastomoses. Due to these facts, use of chemoembolization was casual in the head and neck region to date.

In this study, a new dosage format of cisplatin is introduced combining the advantages described above (high dose, embolization, high response, low toxicity, use of antagonist) without the drawbacks.

## MATERIALS AND METHODS

### Preparation of an aqueous crystal suspension of cisplatin for intra-arterial application

Cisplatin (medac GmbH, Hamburg, Germany) is available in brown glass vials containing a sterile yellowish-white lyophilized powder with 50 mg cisplatin (=pharmacon), 450 mg sodium chloride, 500 mg mannitol and hydrochloric acid (=adjuncts).

The individual patient dosage is 150 mg m^−2^ (Robbins *et al*, 1994). Body surface is calculated by the empirical formula of Du Bois (Du Bois and Du Bois, 1916), limited to a value of 2 m^2^ referring to 300 mg cisplatin.

The cisplatin suspensions were prepared individually at the centralized preparation area for cytotoxic drugs at the Department of Pharmacy at Johann Wolfgang Goethe-University Medical School. They were prepared under aseptic conditions using a vertical laminar airflow work bench (according to DIN 12950) as well as a weight controlled preparation software (Cypro 2.0, ars pharmaceutica Gesellschaft für klinisches Wissenschaftsmanagement und Softwarelösungen mbH, Hamburg, Germany)

The lyophilized drug was reconstituted with 0.9% sodium chloride (Isotone Kochsalzlösung, Braun Melsungen AG, Melsungen, Germany) leading to a yellow 10 ml mixture with a final concentration of 5 mg ml^−1^. The mixture was shaken until the powder was suspended without macroscopically visible clumps. The suspension of the needed number of vials was transferred with a cannula (18G) into two 50 ml disposable sterile syringes (Perfusion Plastipak, Becton Dickinson, Heidelberg, Germany). The syringes were covered for light protection. Although physicochemical data for cisplatin solutions indicates a stability up to 28 days (Trissel, 1998), it was decided to set the maximal expiration time to 8 h at room temperature to limit non reproducible crystal growth. The ready-to-use syringes were labelled with the patients name, dosage, administration day, expiration time and the note ‘please shake before administration’.

### Pharmacological features of the aqueous crystal suspension of cisplatin

**Figure 1:**
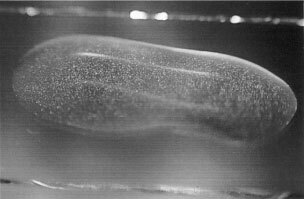
Backlit photography of a drop (12×7 mm) of the aqueous cisplatin suspension on a slide showing the larger crystalline particles.

The preparation method results in a monocomponent, a highly concentrated aqueous suspension of cisplatin with precipitation of crystals. The physicochemical properties (e.g.: Pt^…^Pt distances, molecular vibration analyses) of cisplatin crystals which did form in frozen solutions have been previously described elsewhere (Milburn and Truter, 1966). The stability of the cisplatin complex is pharmacologically assured in a suspension because sodium chloride is solved in a high concentration in this dosage format. The resulting fluid is a 5.4% sodium chloride solution. Hypertonic sodium chloride solutions reportedly do not have effect on pharmacokinetics of cisplatin (medac, 2000). The osmolality is supposedly higher than in an aqueous solution (about 285 mOsm kg^−1^ (McEvoy, 1991)) but cannot be measured exactly because of the presence of crystalline precipitates. Theoretic osmolality of the described suspension as calculated approximately is 2130 mOsm kg^−1^. Microscopic assessment of particle diameters in the aqueous crystal suspension of cisplatin showed rod-shaped crystals measuring 3×8 μm; regular clumping of these crystals formed particles measuring 30×50 μm (Figures 1

and 2

**Figure 2:**
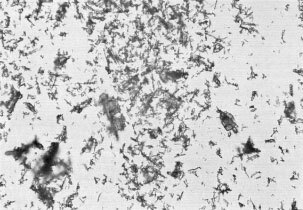
Microscopic photography of the aqueous cisplatin suspension; the crystals are birefringent (200×).

). Ratio of small to large particles was 100/1. In solutions where precipitates did form, redissolution occurred very slowly with warming back to room temperature (Greene *et al*, 1979). At 40°C, the time of redissolution was about 20 to 30 min (Kristjansson *et al*, 1988).

### Patients and study design

In the period between May 2000 and March 2001, 30 Patients with histologically proven, previously untreated primary SCC of the oral cavity and the oropharynx were examined (males/females ratio=22/8; mean age: 60.2±11.5 years; grading II/27, III/3; UICC stages I/2, II/6, III/2, IV/20). The performance status (ECOG (Oken *et al*, 1982)) of the patients, distribution of tumour sizes and regional lymph node classification according to UICC (Sobin and Wittekind, 1997) were noted (Table 1

**Table 1:**
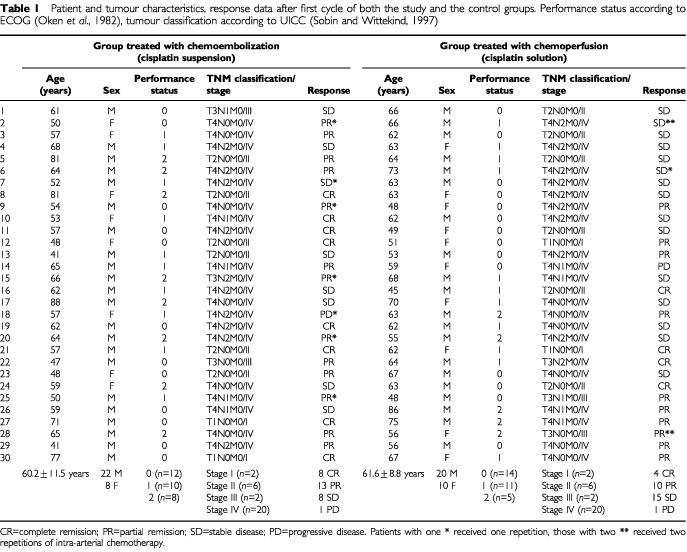
Patient and tumour characteristics, response data after first cycle of both the study and the control groups. Performance status according to ECOG (Oken *et al*, 1982), tumour classification according to UICC (Sobin and Wittekind, 1997)

). Routine pretherapeutic staging procedures included palpation, ultrasound, computed tomography and magnet resonance tomography for diagnostics of neck lymph nodes as well as positron emission tomography for detection of secondary tumours, neck lymph node affection and distant metastases. Positron emission tomography was decisive for staging. Ethical approval was given by the local ethical committee. Informed consent was obtained prior to the onset of chemotherapy and all other therapeutic procedures (e.g.: operation, chemoradiation). The only exclusion criterion was the presentation of a heavy renal insufficiency. All patients took part in a phase II study with a novel concept of integrating induction therapy with intra-arterial cisplatin, radical surgery with neck dissection and combined weekly docetaxel with radiation to the primary site and neck (Kovács *et al*, 2001).

Every patient was treated with one cycle of intra-arterial high-dose chemotherapy in a neoadjuvant setting. Because surgery was considered an important modality of treatment, complete remission was not necessarily aimed at. Regular repetitions of cycles striving at that aim seemed to be too straining for the patients who should undergo a multimodality treatment. In cases of partial remissions after the first cycle, the cycles could be repeated maximally twice, to avoid the development of resistant cell lines. Routine laboratory checks were made on alternate days and compared to pretherapeutic values. The side effects of the cycles according to WHO (Miller *et al*, 1981) were noted. It was decided to stop the study in case of grade III and IV toxicity. Patients were assessed for local response 3–4 weeks later. The dimension of response was assessed clinically (by inspection and palpation) and by CT examination after the first cycle. The remission degrees were defined as follows: CR=complete remission, a complete disappearance of local tumour mass; PR=partial remission, a partial reduction of local tumour mass of more than 50%; SD=stable disease, a partial reduction of local tumour mass of less than 50% or stability of local tumour mass; PD=progressive disease, growth of the tumour >25%. The patients were scheduled for surgery if they were in a general state where the operation should be compatible with life or resection would not threaten vital organs. This judgement was dependent on medical and anaesthesiological examination carried out before the start of any treatment. The patients not treated by surgery were to undergo radiation or chemoradiation therapy.

Thirty patients who had been treated with a cisplatin solution in the period between February 1999 and April 2000 served as control group. Matched pairs were established concerning distribution of pre-treatment tumour and nodal classification. Histologically proven diagnosis in all cases has been previously untreated primary SCC of the oral cavity and the oropharynx. The males/females ratio was 20/10; mean age: 61.6±8.8 years; grading II/26, III/4; UICC stages I/2, II/6, III/2, IV/20. The performance status (ECOG) of the patients also was comparable to the other group (Table 1). They have been accrued for the same multimodality treatment. End points were toxicity and response.

### Management of chemoperfusion

**Figure 3:**
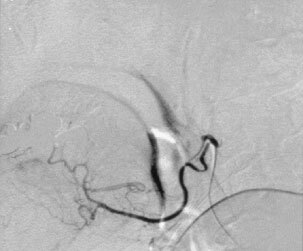
Angiographic film before chemoembolization. Tumour blush in the sublingual area.

At the day of intervention the patients received 74 mg dolasetron and 500 mg prednisolone i.v. (between 0700 and 0800 h Greenwich Mean Time, GMT). A 1.5 l dose of a full electrolyte solution (with 20 mval potassium chloride) was administered subsequently by i.v. infusion over 2 h. Transcutaneous catheterization of the right femoral artery was carried out subsequently using a 4-french catheter containing a coaxial micro-catheter. Superselective visualization of the tumour-feeding vessel and assessment of its volume capacity using fluoroscopy and a contrast medium were performed. It never has been necessary to perform a provocation test because angiography in these areas usually reveals enough information. Then, 150 mg m^−2^ cisplatin (maximal absolute dose 300 mg, lowest dose 225 mg) suspended as mentioned above (maximal amount of fluid: 60 ml, smallest amount 45 ml) was infused with controlled pressure via a hand-held syringe. In all cases, stasis of flow inside the peripheral capillaries of the tumour could be noticed via fluoroscopy (Figures 3

and 4

**Figure 4:**
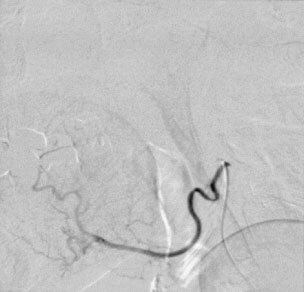
Angiographic film following chemoembolization using the aqueous crystal suspension of cisplatin via the lingual artery. Occlusion of the tumour-feeding vessels.

). For analgesia, 0.1 to 0.4 mg fentanyl and 1 to 2.5 mg droperidol were delivered i.v. before start of therapy. In cases of toothache, mainly observed during perfusion of the maxillary artery, 1 ml of a 1 : 10 diluted 1% lidocain-HCl injection into the perfused artery was able to block the pain. With a delay of 10 s, an i.v. infusion of 9 g m^−2^ sodium thiosulphate was given in parallel. After the end of the chemoperfusion no more sodium thiosulphate was given. Application of the cisplatin suspension was finished a maximum of 3 h after preparation. One thousand millilitres of full electrolyte solution with 20 mval potassium chloride was infused again (i.v. over 5 h, subsequent to treatment). Administration of 500 mg prednisolone i.v. was repeated between 1600 and 1800 h GMT, with further repetition over a maximum of 3 days depending on local swelling. The day after intervention, the patients were hyperhydrated with 3000 ml of a two-third electrolyte solution, received a thrombosis prophylaxis with heparin S.C. (and dolasetron i.v., if necessary). Routine analgesia was given with metamizol drops.

In the control group treated with a cisplatin solution, management was very similar. No prednisolone was given, 150 mg m^−2^ cisplatin was dissolved in 500 ml 0.9% saline solution and was perfused with controlled pressure (2 ml s^−1^). Perfusion took about 5 min.

## RESULTS

Chemoembolization was carried out 37 times in the 30 patients. In five patients, there has been one repetition according to the study plan; in patient no. 7 the tumour was so large that good remission of the embolized part had to be assessed as overall stable disease and repetition seemed to be promising, and in the only patient with progression a desperate attempt was made by repetition. Duration of injection at the end of neuroradiologic intervention was between 60 and 90 s. Toleration of chemoperfusion was good and the dosage of analgetics sufficient. There have been two complications of transcutaneous femoral catheterization: 6 days after intervention, a patient (no. 11) suffered from a scrotal haematoma, which was treated conservatively and experienced resorption after 15 days; one patient (no. 28) showed signs of embolism of the left leg at the day of intervention. For three years since, he had an aortofemoral prosthesis. Treatment with heparine was quickly successful.

In the control group, there have been 35 interventions in 30 patients. In three patients, there has been repetition of cycles (once two cycles, twice three cycles). The two patients with three cycles refused surgery and radiation; one of them with stable disease died due to heart failure, the other patient with partial remission is alive and well 9 months after the end of treatment. In one patient (no. 27), there has been an arterial occlusion in the right leg which could be treated successfully without sequelae.

Remissions after one cycle are shown in Table 1. The response rate (CR+PR) in the study group was 21/30 (70%). The tumours of patients classified as stable disease showed reduction of tumor mass of about 30%, large areas of necrosis and regressive alterations in CT. The response rate in the control group was much less pronounced (14/30=46.7%). Tumours classified as stable disease showed less regressive alterations in CT.

The systemic and local side-effects of both groups are listed in Table 2

**Table 2:**
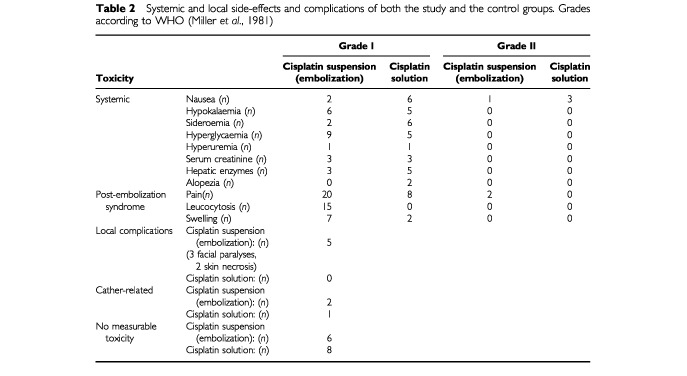
Systemic and local side-effects and complications of both the study and the control groups. Grades according to WHO (Miller *et al*, 1981)

. In comparison with the regime using a cisplatin solution, there have been less nausea, sideroemia, increase of hepatic enzymes and no alopezia in the study group. Slight hyperglycaemia up to 140 mg dl^−1^ vanished after 6 days. Systemic toxicity was very low in both groups. There have been no grade III or IV toxicities at all. Six and eight patients, respectively, showed no measurable side-effects.

The local symptoms of pain and swelling combined with a non-febrile leucocytosis (up to 19 000 mm^3^) were similar to the known ‘post-embolization syndrome’. These symptoms were seen mainly or exclusively in the study group. Swelling of the cheek and the tongue was soft and vanished after 10 days. If swallowing was impaired, a gastric tube secured enteral nourishment. Moderate pain (grade II) was treated with metamizol and tramadol drops and, if painful superinfection in swollen epidermolytic areas of the oral mucosa was noticed, an oral antibiotic (clindamycine) was prescribed for 5 days. Leukocytosis was not treated and normalized after 10 days. Ward stay lasted between 3 and 17 days (mean: 9.1 days) which was necessary for financing this interventional procedure under the conditions of German hospital regulations. Although the patients of the control group showed much less local symptoms, ward stay had to be extended up to 6 days for the same reason.

There have been five local complications in the study group resulting from embolization of branches of tumour-feeding vessels as perfusion of the deep temporal arteries during embolization of the right maxillary artery (patient no. 3). A circumscribed necrosis in the temporal muscle with temporary limitation of mouth opening and a paralysis of the facial nerve both resulted. Limitation was over 4 weeks later, recovery of nerve function took 3 months. Two other patients (nos. 17 and 24) suffered from temporary facial paralysis due to perfusion of the geniculate ganglion via the medial meningeal artery. The tumours had to be embolized via the maxillary artery in both cases. In two patients (nos. 10 and 11) skin areas of the cheek and the chin were additionally embolized and developed necrosis. This did not cause harm to the patients because these skin areas had to be resected during the operation in healthy margins.

In the study group, 21 patients were operated on. Patients classified N0 underwent elective neck-dissection, patients classified N1–3 modified radical neck-dissection. In three specimens, there has been evidence of complete histological remission (pT0), very small tumour tissue could be found in five specimens (pT1). There have been one pT2 and 12 pT4 specimens. Two patients refused further treatment, three patients were radiated after chemoembolization, four other patients were in too bad general condition for either surgery or radiation. To date, there has been one patient (no. 1) with distant metastasis to the vertebra 2 months after the end of treatment who was operated on for palliation. Four patients are dead. One patient (no. 17) died 1 week after the operation due to heart failure, two patients (nos. 5 and 6) died several days after the end of radiation, one patient (no. 15) died due to extreme tumour cachexia.

In the control group, 24 patients were operated on with the same criteria for neck surgery. Twice, there has been complete histological remission (pT0), five times pT1, seven times pT2, twice pT3 and eight times pT4. One patient was radiated after chemotherapy, two patients refused further treatment, three patients were in too bad a general condition for either surgery or radiation. There have been two local relapses and one regional relapse to date. One patient (no. 11) could be treated successfully by salvage operation, one patient (no. 19) is treated to date with low-dose methotrexate for palliation, one patient (no. 6) died 10 months after the end of treatment. There have been three secondary tumours (patient no. 10 with lung cancer, patient no. 14 with a maxillary tumour, and patient no. 28 with a hypopharyngeal tumour). Patient no. 10 died 12 months after the end of treatment, the others are treated to date. One patient (no. 2) died after the third chemotherapy cycle, one patient (no. 18) died 12 days after the operation, both due to heart failure. In sum, four patients are dead to date.

## DISCUSSION

Intra-arterial chemoembolization was mainly used as treatment of primary hepatocellular carcinomas and liver metastases. To date, embolization was achieved either with encoated drugs (microcapsules) (Kato *et al*, 1996) or with concomitant delivery of embolizing substances like polyvinyl alcohol, lipiodol, albumine, degradable starch microspheres using mixtures, sometimes with several drugs (Tellez *et al*, 1998; Vogl *et al*, 2000; Yamamoto *et al*, 2000). These protocols have certain advantages: microcapsules provide good reproducibility of the product with good control of particle diameter, and a pharmacologically adjusted time of drug release. The embolizing substances occlude the tumour-feeding vessel for a certain time and stop intratumoural flow before being dissolved. These advantages compete with serious drawbacks: production of microcapsules is a complicated and expensive technical process, often necessitating 900% more encoating material than drug. The patient is stressed with additional substances which is also a drawback of the combination of several drugs (polychemotherapy) justified mainly in palliative treatment. In the case of auxiliary substances (it may be a viscous suspension like lipiodol or different larger microparticles), there is also the problem of production taking much costs, time, and effort. It is obvious that any embolizing agent would be extremely dangerous in the head and neck area. Anastomoses to the ophthalmic artery and arteries feeding nerval structures must not be occluded, not even a short time. Even short complete occlusion of vessels like the facial or lingual artery would have the risk of necrosis of important organs which is intolerable. The amount of the embolizing agent cannot be completely assessed before the action of delivery because the exact diameter of the perfused vessels and capillaries is not known and delivery has to be continued until occlusion is achieved. In the head and neck area, complete occlusion should be aimed at very cautiously. These are the reasons why chemoembolization was used rarely in the past. According to the body of literature, only 54 head and neck SCC patients were treated using embolization protocols in Japan and China in a period of more than 14 years (Okamoto *et al*, 1985, 1986; Kato *et al*, 1996; Tomura *et al*, 1996; Konno *et al*, 1998; Tomura *et al*, 1998; Li *et al*, 1999).

In contrast, the new technique presented has many principal advantages. It provides a monocomponent (cisplatin as made available by the producer) which serves as microembolizing agent itself. No additional pharmacon is necessary, preparation is easy. The crystalline precipitates have a rather regular diameter of 3×8 μm and agglomerate to maximum 30×50 μm particles, thereby allowing periphery embolization of intratumoural capillaries but vessels with a larger diameter (mainly the facial or lingual artery) are spared. The time of dissolution of these crystals in the arterial flow at body temperature is likely to be about 20 to 30 min as having been assessed at 40°C (Kristjansson *et al*, 1988). The demonstrated tumour response rate is a clear indicator of better effect than achieved by a solution. It can be subsumed that the reason for this rate is not only the higher concentration of the drug in the suspension but the presumed slower intratumoural dissolution of the crystals.

Superselective administration of the agent into the tumour-feeding vessel is mandatory. Larger vessels normally cannot be occluded. The observed accidental occlusion of the deep temporal arteries must have been caused by their individual small diameter.

Not only is the response better but the systemic acute toxicity is lower. This may have the following reasons. A higher intratumoural concentration resulting from a higher first-pass or trapping effect may be likely. A technical detail contributes to the favourable findings of higher response and less systemic toxicity. Intraarterial delivery of the smaller amount of fluid (maximum 60 ml aqueous cisplatin suspension) using a hand-held syringe results in better time management with consideration of the parallel intravenous infusion of sodium thiosulfate. The large amount of fluid used for the cisplatin solution (500 ml) sometimes led to spasms of the vessel and interruptions of perfusion. Duration of administration was longer (5 min), the pumps used provided a standardized flow but were clumsy in operation. Now the capacity of the vessel could be assessed by hand-controlled injection of saline first, followed by injection of the suspension with the same pressure. The injection could be executed quickly in less than 2 min without reflux of blood and possibly sodium thiosulphate, a good flow could be achieved with full exposure of the tumour bed to cisplatin. Sodium thiosulphate has reached the periphery when cisplatin arrived there.

The local side-effects could easily be overcome and posed no problems for maxillofacial routine care.

There are no presently available multi-agent chemotherapies with comparable high response rates after one cycle and similar low rates of acute side-effects. Main problem of the presented method is potential facial paralysis which may occur in case of perfusion of the maxillary artery or the external carotid artery. This can be avoided by excluding patients where this is expected and using the cisplatin solution instead where no embolizing effect is present.

To our knowledge, the only explicit attempts reported in literature, to suspend cisplatin in high concentration in saline were carried out by a Japanese group (Kawakami *et al*, 1988; Ono *et al*, 1989). They needed a control for investigating the effects of a cisplatin-lipiodol suspension on the rat liver. As is common knowledge today, they found a higher affinity of lipiodol to liver tissue. The potential of the aqueous cisplatin suspension with its precipitation of crystals was not recognized. Tsujimoto *et al* (1993, 1996) used specially manufactured cisplatin microcrystals of less than 500 μm in diameter suspended in a mixture of sesame oil and lipiodol. They investigated the toxicity of intraperitoneal administration of that suspension in mice. The toxicity was equal to or less than with a regular cisplatin solution. For preparation of the infusion of a cisplatin solution, the manufacturer recommends diluting the cisplatin dose to a maximal concentration of 1 mg ml^−1^ (Trissel, 1998). At concentrations higher than 1 mg ml^−1^ precipitation occurs and cisplatin is not dissolved totally. The use of a concentrated injection seemed justified for two reasons: the absolute dose of cisplatin remained the same as in the well established settings of intra-arterial chemotherapy of more than 300 head and neck cancer patients (Kovács *et al*, 1999; Robbins *et al*, 2000), and highly concentrated mixtures of cisplatin with embolizing agents with same or larger particle diameters were used for decades in the treatment of patients with liver cancer and metastases (Tellez *et al*, 1998; Yamamoto *et al*, 2000). The high concentrations up to 10 mg cisplatin in 1 ml lipiodol (Yamamoto *et al*, 2000) or other embolizing agents were empirically motivated and had no pharmacological foundation. Dosages were much lower (maximum 100 mg m^−2^) than in the present study. Using the method described in this study, the monocomponent as provided by the manufacturer could be used as embolizing pharmacon without additional agents. Moreover, the required small volume for rapid injection was achieved. Use in the head and neck area proved to be feasible.

The combination of anti-neoplastic effect with microembolizing features offered by the same anti-neoplastic pharmacon was made possible by dispersing that pharmacon in an aqueous crystal suspension. This new principle called ‘monocomponent chemoembolization’ showed the first good results in routine intra-arterial chemoembolization of oral cancer patients and will be continued. It may also be investigated in other indications.
